# Anthropological Research Study of Migrants at the First Aid and Reception Center (CPSA) of Lampedusa

**DOI:** 10.3390/ijerph19095337

**Published:** 2022-04-27

**Authors:** Maria Concetta Segneri, Anteo Di Napoli, Gianfranco Costanzo, Concetta Mirisola, Andrea Cavani, Miriam Castaldo

**Affiliations:** 1 Medical Anthropological Unit, Department of Mental Health, National Institute for Health, Migration and Poverty (INMP), 00153 Rome, Italy; miriam.castaldo@inmp.it; 2 Department of Epidemiology, National Institute for Health, Migration and Poverty (INMP), 00153 Rome, Italy; anteo.dinapoli@inmp.it; 3 Health Directorate, National Institute for Health, Migration and Poverty (INMP), 00153 Rome, Italy; gianfranco.costanzo@inmp.it; 4 INMP Directorate, National Institute for Health, Migration and Poverty (INMP), 00153 Rome, Italy; concetta.mirisola@inmp.it; 5 Scientific Coordination Unit, National Institute for Health, Migration and Poverty (INMP), 00153 Rome, Italy; andrea.cavani@inmp.it

**Keywords:** forced migration, violence and torture, journey, medical anthropology

## Abstract

A medical anthropology research study was conducted in 2015 at the First Aid and Reception Center (CPSA) on the island of Lampedusa (Italy) as part of a larger health project carried out by the National Institute for Health, Migration and Poverty (INMP) in Rome. The study investigated the health conditions of migrants at the moment of their departure and on arrival, their migration journey, and their life plans and expectations for the future. The ethnographic method adopted for the study was based on participant observation and on data collection by means of a semi-structured interview (51 items simultaneously translated by cultural mediators into Tigrinya, Arabic, English, and French). Interviewed were 112 adults (82 men and 30 women) from the Gulf of Guinea and the Horn of Africa. The cooccurrence of forced migration and economic concerns was confirmed; violence and torture were constants throughout the migration journey in 81% of cases. Ethnographic data detailed the timing, countries, settings, perpetrators, and types of violence endured. A combination of qualitative and quantitative findings can both facilitate the identification of fragile health conditions and support clinicians in the diagnostic, therapeutic, and rehabilitation pathways. These data illustrate the importance and feasibility of multidisciplinary collaboration even in emergency contexts.

## 1. Introduction

Since the late 1990s, the island of Lampedusa, Italy, has become one of the main destinations of African migration routes in the Mediterranean [1,2].

From 1 January to 1 October 2015, 136,432 migrants arrived on the coasts of Italy; of these, 19,019 arrived on Lampedusa. Although there were fewer landings than in 2014 (−7.4%) [3], in 2015, Italy had the second-highest number of migrant arrivals in Europe, after Greece [4]. The countries of origin included Eritrea (27%), Nigeria (13%), Somalia (8%), Sudan (6%), and Syria (5%) [3].

That same year, in an effort to better manage the migration flows to Italy and Greece, the European Commission introduced a new reception system for migrants based on hotspots to replace the First Aid and Reception Centers (CPSA) [5].

Once disembarked, the migrants were identified and underwent a health assessment by medical personnel of the Italian National Health Service (NHS) and international organizations (NGOs), as well as by the hotspot staff [4,6,7,8].

When the anthropological research study was carried out, the following were present in the center: the army; the police; international agencies (UNHCR, IOM, and Save the Children); the Italian Red Cross; the cooperative managing the services for migrants inside the center; and the Italian NHS.

Health assessments were supported by a rotating National Institute for Health, Migration and Poverty (INMP) multidisciplinary team composed of infectious diseases specialists, dermatologists, psychologists, cultural mediators, and medical anthropologists. The latter were included on the team to ensure that a holistic approach was provided to migrants and to support clinicians in their choice of the most appropriate therapeutic pathways.

Overland migration [9,10,11] and forced migration [12,13] are highly traumatic [14] and causes of certain pathologies [15,16,17,18].

Based on the assumption that migration may have a significant impact on health, the strategic position of Lampedusa allows for the collection of information on a migration journey recently ended. The main value of the study is the production of quali- and quantitative data that can both facilitate the identification of fragile health conditions in migrants and support clinicians in the diagnostic, therapeutic, and rehabilitation pathways. Furthermore, the findings show that multidisciplinary collaboration is possible even in emergency contexts.

The purpose of this study was to describe the characteristics of violence inflicted on migrants, the migration routes taken, and their health status before and after the migration pathway.

## 2. Literature Review

In order to carry out the anthropological research, a review of the international literature was conducted.

First, we tried to understand the migration flows arriving in Europe by sea—in particular, whether the migrants arriving on the Italian, Greek, and Spanish coasts came from the same countries and whether there were significant incidences regarding gender and age. Italian, European, and international sources revealed that migrants arriving on European shores were mainly men who came from roughly the same African and Middle Eastern countries [3,5,15,19,20,21]. We then investigated whether those arriving on European shores had travelled with valid identity documents and regular visas throughout their journey, how many migrants sought asylum on arrival, and how many were actually entitled to international protection. In fact, sources indicate that migrants arriving in Europe by sea travelled without documents, relying on human traffickers, that all of them were seeking asylum, and that most of them were actually entitled to it [10,14]. Some were fleeing from overt conflicts, and others were fleeing from conditions of extreme socioeconomic precariousness caused by low-intensity conflicts that had lasted for decades. For this reason, the literature consulted showed that, for several years now, European governmental bodies responsible for assessing asylum applications have struggled to distinguish clearly between conditions of forced migration and what are called economic conditions [15,16].

We then sought to understand the health status of migrants who landed on European shores, investigating and focusing on the trauma they had suffered from violence and torture. This focus was important, because studies have reported that migrants travelling without documents through human traffickers and fleeing conflict have often had traumatic experiences [6,7,8,9,11,12,17,18,22,23,24]. Trovato et al. [8] effectively summarized the self-reported health conditions of migrants upon their arrival in Italy at the port of Augusta. The impact of these traumas on their health was considered by clinicians to be so significant that having more detailed information about the trauma could benefit health interventions. This was highlighted both by Arsenijević et al. [6] and by Napolitano et al. [18], although in reference to different contexts—the Balkans Europe and Italy, respectively. These studies also emphasized the importance of using multidisciplinary teams and both quantitative (typical of biomedicine) and qualitative (typical of psychology and other human sciences) methods of investigation of these populations in primary and secondary reception centers [10,25,26,27]. For this reason, Schilling et al. [25], focusing on the health conditions of migrants, emphasized the need to calibrate healthcare on the basis of the multiple traumatic experiences reported by patients. The social use of medical anthropology in multidisciplinary and multiprofessional teams can intervene by leveraging the practices and approaches to the care of migrants through targeted returns of its research, as is the case of the present study [28,29]. In addition, we conducted an analysis on the anthropological research conducted at the primary and secondary reception centers in order to more deeply investigate and, especially, to collect qualitative data using the ethnographic method in order to use them in diagnostic processes and therapeutic pathways. The sources showed that the ethnographic surveys carried out had focused mainly on the impact of European legislation and reception systems on the recognition of asylum and on migrants’ health conditions in general [30,31,32,33,34]. What emerged from the observations of psychologists and psychiatrists working with migrants arriving by sea and asylum seekers, however, was the usefulness of more in-depth, more precise knowledge of traumatic experiences [4,6,7,8,9,12,18], which would clearly reduce possible the retraumatization [27,35,36]. Anthropological studies were also considered regarding the right to health exercised through international protection and the right to stay in the country of arrival exercised through a sick body, visible injuries, evidence of suffering that conceal the individual and his/her history, making him/her a passive subject, worthy of becoming a citizen by right only if and only as a sick person, an object of care and compassion [37,38,39,40,41,42,43].

Lastly, as the aim of our research was to produce a picture of trauma that was as complete as possible in terms of the details chosen (country, type, place, perpetrator of the violence, and the torture suffered), sources regarding the collection of qualitative data on trauma, causes of migration, migration routes, and the perception of their health status from their country of origin to arrival in Lampedusa were studied in depth.

These analyses made it possible to develop codes and classifications that could be easily understood and used by health personnel in first aid settings, starting from the classifications of violence and torture as described in the Istanbul Protocol [44], then arriving at a synthesis adopted by various studies [4,7,12,16,17,23,25,45]. The analysis of the literature focused on studies that observed violence and torture suffered from the beginning to the end of the migration journey, detailing the country, type, place, and perpetrator. Crepet et al. [4], for example, using tables to distinguish the types of violence suffered during the journey, emphasized above all the psychological aspects. Bouhenia et al. [12], Ben Farhat et al. [7], and Nakash et al. [17], on the other hand, although referring to different contexts, reported a variety of data (presented in tables) connecting the various stages of the migration route to the various types of violence experienced. Mainwaring and Brigden [16] also included the subjective consequences of the violence suffered in terms of social relationships and cultural identity by observing the places where the violence was inflicted. The same attention was paid to causes of migration, routes, and health perceptions. This work took into account the international political and, especially, biomedical construction of these classifications and their use in biomedicine at the places where migrants are received [37,38,41,42,46].

## 3. Materials and Methods

### 3.1. Structure and Timeline

From 4 May to 20 September 2015, the INMP multidisciplinary team provided specialized assistance to the clinical teams working inside the CPSA in Lampedusa by virtue of their specific experience in the diagnosis and treatment of the health problems of migrants.

In this context, medical anthropologists conducted this anthropological research study inside the CPSA with the support of cultural mediators.

The study compared the health conditions of migrants at the time of their departure from their country of origin and at the moment of disembarking to analyze the impact of the migration journey on their psychological and physical health. These migrants’ life plans and future expectations were also investigated. This anthropological research study was divided into three phases. In the first, an analysis of the research field and its actors was carried out: observations of the migrants themselves and of those providing primary care, services, and security were conducted over the course of 15 days at the CPSA. In the second phase, research tools (a semi-structured interview, a database, and field notes) were developed and tested. In the third, qualitative data collected were analyzed, and the most suitable coding method was chosen to guide the clinical action. The latter requirement of being useful to the clinic has led to a greater emphasis on descriptive data than reflective data, simplifying the results using specific categorizations.

### 3.2. Tools and Recruitment

Included in the study were those migrants who gave their informed consent to participate. The research objectives were explained to the migrants by providing brief information to allow mutual understanding. It was made explicit that, because they would be asked to recount experiences of great emotional impact, they were free to interrupt or postpone the interview if it became too painful so as to avoid possible retraumatization [27,35,36]. Additionally, an attempt was made to explain the role of the medical anthropologist, since this profession is not well-known in many migrants’ context of origin.

Ethnographic tools enabled us to collect qualitative data on the traumatic events the participants faced during their overland migration journey, allowing us to reconstruct a timeline of where these events occurred (both short detention and long-term imprisonment), who the perpetrators of the violence were (the military and the prison police), and the type of violence inflicted. As reported in the previously mentioned studies conducted on migrants’ arrival in Italy [4,8], qualitative tools are the most appropriate to investigate migration trauma in depth. Indeed, the collected ethnographic data gave us detailed information on some specific aspects of violence against migrants

The interview was written in Italian (Appendix A) and was simultaneously translated into four languages by cultural mediators: Tigrinya, Arabic, English, and French. A cultural mediator is a professional who facilitates the communication (including interpretation) between people speaking different languages and with different sociocultural backgrounds Thanks to him, it was possible to take into account those aspects that influence the collection of qualitative data, such as the interviewees’ sociocultural references, their complex general condition at the time of the interview, and the context where the interview was conducted. However, cultural mediators were excluded from some interviews when requested by the interviewee.

The interview lasted between 45 min and 3 h. In some cases, the interviews were conducted in phases to accommodate the center’s activities (e.g., medical check-ups, meals, distribution of clothing and personal hygiene products, fingerprinting, and so on). Sometimes, after these interruptions, it was not possible to resume and/or conclude the interviews.

### 3.3. Collection, Classification, and Analysis of Data

The field research started with the collection of qualitative data. The interview (Appendix A) included 51 items: personal details; reasons for migration; travel details (planning, costs, means of transportation, accommodations, and autonomy); migration stages and routes; health status (before and after departure from country of origin); violence and torture endured (country of origin and at what point of the journey); previous migration of family members; any practical support received, life plans and future expectations.

The same interview was conducted for the men, women, and children who participated in the study. However, as the qualitative data on the traumatic aspects of migration did not differ between these groups of participants in any relevant way, they are not reported in the Results.

The research focused on any violence and torture experienced by the participants, analyzing when it was inflicted (initial or final stages of the journey); in which country; in what setting (military checkpoints, police stations, and prisons); by who (the military, the prison police, and traffickers); and what kind (following the 21 categories defined by the Istanbul Protocol) [44].

The study also analyzed the participants’ self-perceived health at the time of their departure from their country of origin and whether they felt it had changed during their migration journey.

The data obtained during the interviews on experiences of violence and on self-perceived health were classified according to the literature.

In particular, starting from 21 subgroups of violence and torture [4,7,12,16,17,23,25,45] (Table 1), we defined the six types to which migrants are most exposed (physical, sexual, and psychological abuse; detention conditions; threats; and sensory deprivation).

Moreover, the reasons for migration and the routes were categorized on the basis of previous studies [19,20,21].

The reasons for migration were categorized into main (international protection and economic factors) and specific, based on the 15 types reported by the study participants (Table 2). Where participants were potential applicants for international protection, possible biases reported in the literature during the narratives provided by migrants for asylum recognition in Europe were taken into account [37,38,39,40,41,42,43]. Studies on the control and confinement system to which migrants are subjected upon arrival in Europe were also considered [30,31,32,33,34].

Furthermore, starting from countries crossed during the entire trip, six main routes were categorized: North Africa, Central-North Africa, East-North Africa, West-Central-North Africa, West-North Africa, and Asia-Africa.

The last step of the study was to analyze the data collected, which are presented as absolute and relative frequency tables. The method of analysis chosen favored descriptive data [30] over reflective data, resulting from a critical reading of the context and the sociopolitical dynamics observed in the place where the study was carried out [31,32,33,34]. This choice stemmed from the need to distinguish between, on the one hand, what could be immediately useful to the clinic in orienting its work and, on the other, what could be important to report to all the actors involved during the primary reception of populations seeking international protection. In fact, the actors involved were different (as mentioned above); they had different roles and functions in the place where the study was carried out, and above all, there were political balances between them to which we had to pay great attention. All the actors collaborated in making the roles and functions clear and respected, and above all, there had to be no overlap in the institutional mandate of each. In this study, only the outcome of the analysis of the descriptive data collected has been reported.

## 4. Results

From 4 May to 20 September 2015, 112 people were interviewed: 82 men (73.2%) and 30 women (26.8%), with an average age of 24.4 ± 6.7 years (range 10–46).

Most of the interviewees came from six African countries in the Gulf of Guinea and the Horn of Africa: Eritrea (27 people, 24.1%), Nigeria (26, 23.2%), Sudan (9, 8.0%), Gambia (8, 7.1%), and Ghana (6, 5.4%), as well as from Syria (7, 6.3%). Most (75%) were not married at the time of the interview.

The participants’ education levels were mostly low-medium: 38.4% had primary education (6–8 years of study), 20.5% first-level secondary education (9–13 years of study), and 17.9% second-level secondary education (14–19 years of study), with 13.4% having only up to 5 years of education. With regards to the reasons for migrating from their country of origin, 61.6% reported international protection, while 58.9% indicated economic factors. It is interesting that 60.6% of the participants did not recognize any critical factors in their country of origin, for example, low-intensity conflict, as the indirect cause of their migration.

The participants reported having taken one of 24 different routes, across one to six countries, to reach Libya, the last stop before taking a boat to Lampedusa (Table 3). The main routes were North Africa (22.2%), Central-North Africa (24.2%), East-North Africa (30.3%), West-Central-North Africa (5.1%), and West-North Africa (12.1%), while another route starting from Asian countries reached Libya through Africa (6.1%).

Table 4 shows the self-perceived health status of migrants by the stage of the migration route. A deterioration in the self-perceived health status was observed in 81.3% of participants during the journey compared to that in their country of origin, with an improvement in 59.8% of subjects upon arrival at the CPSA in Lampedusa. With regards to the latter, the strong recovery in health perceived by participants upon arrival at the Center could also depend on the perception of safety acquired during the first aid and reception procedures. A deterioration in self-perceived health status was reported by participants in all cases if the macro area route was Asia-Africa, 90.0% of cases for North Africa, 83.3% for West-North Africa, 78.6% for East-North Africa, 66.7% for Central-North Africa, and 60.0% of cases for West-Central-North Africa.

About half of the migrants self-reported a poor health status. Among those participants who requested medical attention at the center, 64.8% reported health problems resulting from their migration journey, 21.1% showed symptoms from a nonspecific cause, and 12.7% already had symptoms in their country of origin (Table 5).

Regarding violence, 75.9% of participants reported having experienced at least one form of violence (out of six types) since leaving their country of origin.

While only four participants had been in prison in their country of origin, most participants (71.8%) had been imprisoned during the migration journey, especially in Libya (90.8%): in a prison (14.6%), in some type of detention facility (50.0%), or in both (7.3%).

Among the participants, 33.0% experienced violence and torture throughout the migration journey: 18.8% suffered violence and torture while detained, either at police stations or at the border facilities, and 49.1% in a prison or other detention facility. Only one person reported having experienced violence and torture both in his country of origin and during the migration journey, prior to emigrating to Italy via Libya.

The most common forms of violence and torture were physical (50.89%), psychological (48.21%), related to detention conditions (33.04%), and threats (25.89%) (Table 6), and 81% of the participants declared that the main perpetrators of the violence and torture were the military and the prison police.

The last data point collected analyzed the participants’ migration plans and their reflections on their success. About 80% of the participants planned to conclude their migration by remaining in Italy (33.8%) or by moving to another European country where they already had relatives—for example, Germany (22.1%), Sweden (14.3%), or the United Kingdom (11.7%). Further, 42.9% declared they had planned to ask their relatives for initial support upon arrival; in fact, 40.2% had relatives who had already emigrated to Europe. There were also those who had initially planned to stay in Libya (13.0%) but, once there, decided to continue their journey because of the abominable conditions they had faced. Finally, 71.0% did not rule out the possibility of one day returning to their country of origin, where they had left their family (97.3%). Only 23.2% of the participants had arrived at the CPSA with their family.

## 5. Discussion

This study showed that the participants had repeatedly undergone violence and torture during their migration experience, thus confirming the high degree of trauma caused by the journey reported in the literature. Migrants are not automatically vulnerable to poor health outcomes. It is the conditions associated with different phases of the migration journey (pre-migration, transit, arrival, and return) that may negatively or positively affect health [14]. By combining quantitative and qualitative data and comparing them with the categorizations codified by studies in the field, the current study identified a “methodological rigor in analyzing systematic exposure to such trauma” to investigate in detail the trauma produced by the migration journey, as other studies have done [17].

Moreover, this study analyzed the migration journey from start to finish based on what was told by the participants. This complex experience (social, economic, and political) involves multiple actors and dynamics that impact the subjectivities of the people and models of sociality with performative power [16,39,41,43]. Finally, based on previous research [6,7,12,18] analyzing violence during the migration journey, this study paid close attention to the following: when the violence occurred (initial, intermediate, or final stage of the journey), in which country and setting, who the perpetrators were, and what type of violence was inflicted.

One study [6] analyzed the “systematic and organized nature” of the violence perpetrated on migrants upon arrival in European countries, while another [12] examined the violence inflicted on migrants in their country of origin before departure. Our study focused on the violence suffered by migrants from the moment of their departure to their arrival, looking for any common characteristics among the individual experiences reported. In line with the previously mentioned studies, this study showed that multiple episodes of violence and torture took place and that they were mainly physical and psychological. Violence and torture were inflicted mainly during the migration journey and, specifically, in the last stage, Libya, where the participants underwent violence mainly in three settings: prisons or other detention facilities, border facilities, and police stations [10,12].

Thanks to the ethnographic method’s ability to bring out the implicit present in the accounts of people’s life experiences [30], the results also showed that 60.55% of the participants were not able to recognize the overall causes that had forced them to leave their own country; indeed, most identified the most striking and immediate factors as the reason for their departure. Moreover, 42.86% expected support from relatives who had previously immigrated to Europe. This demonstrates the existence of an “emigration practice” that these countries of origin have established over time. These data confirm the results of previous studies about forced migration; when we talk about countries characterized by low-intensity conflicts, we can state that it is economic reasons that have caused diasporas, but they have resulted from persistent political and social instability.

An explicit example of this was given by the study participants from Eritrea (24.1%). These people, fleeing the military regime established in 1993 by President Isaias Afewerki, often cited poverty as a factor in their leaving their country. However, an in-depth examination of what was happening in their home contexts revealed a much more complex picture, based on policies of repression, violence, and the suspension of human rights, which, for generations, had driven the local population to migrate to Northern Europe. In fact, the widespread impediments to personal freedom and the compulsory military service for men and women from the age of 16 for an indefinite period did not allow the population to earn enough for minimum family needs, forcing them into forms of forced labor, as well as exposing them to fierce repression if they abandoned military service [47]. Over time, all this gave rise to a diaspora towards Scandinavian countries, establishing, at the same time, a community emigration tradition.

In fact, when referring to how “mixed migration” develops, some authors talk about its “piecemeal nature”, which causes a “humanitarian catastrophe” because of the episodes of violence and death during transnational displacement [15,16].

Despite the subjective narrative details, the data confirmed that all participants experienced violence and torture during their overland migration journey, regardless of their gender, age, country of origin, migration route, or cause of migration.

## 6. Strengths and Limitations

This study has several strengths, the first being that it provides qualitative ethnographic data, which are usually difficult to collect; the authorities responsible for first aid centers for migrants rarely allow this kind of study to be conducted in this setting because of the associated confidentiality requirements. In addition, the data collected are valuable thanks to the methodological effort carried out, which make them more suitable for clinical use. Finally, the study was carried out at one of the first aid centers for migrants closest to a point of departure, for example, Libya. This means that the data were collected from a privileged position both in terms of timing (the migrants’ arrival in Italy) and in terms of their uniqueness (in many cases, this was the first time the participants talked about their migration journey).

The qualitative data collected allowed us to learn even more about migration (causes of migration, violence and torture suffered during the journey, and migrants’ self-perceived health). Additionally, the temporal proximity to the reported traumatic events was effective, because the participants’ memories were more vivid. Ultimately, the collection of these specific ethnographic data had a notable impact on the investigators collecting the data because of the narrative content (the participants recounted that they had escaped death and had survived very dramatic experiences).

This anthropological research study is innovative, because it reports uncommon qualitative data on migrants in a First Aid Center regarding issues that are widely discussed in the literature, i.e., the violence and torture migrants suffer during the migration journey.

The advantage of collecting these particular data on arrival in the host country can be appreciated during the initial health assessment, when each migrant’s main vulnerabilities are identified and the most appropriate treatment is chosen [4,8]. In fact, the ethnographic method makes it possible to study, in depth, multiple aspects of the life experiences that can be useful for a clinical anamnesis carried out in emergency contexts where the intervention priority aims at the health safety of large groups of people (e.g., from infectious diseases). For these reasons, this in-depth anthropological analysis is also meaningful during the delicate, complex phase of the secondary reception [9,10,18]. In fact, trauma involves immediate and long-term consequences on migrant health. Moreover, migrants may encounter circumstances that could cause retraumatization. For example, this happens when health interventions do not consider the fact that the social patterns of health and disease are defined by the individual’s sociocultural horizon. This study has some limitations, the first related to the limited number of participants in the study compared to those who we would have liked to interview. The limit of the number of participants arises, because this anthropological study was carried out in support of clinical activity and, therefore, to respond (in the most complete way possible) to an immediate operational need. It was a study of applied anthropology, where “applied” in this case means improvement of clinical actions, and the clinic in emergencies (such as the one where the study was conducted) intervenes on a large number of people, much greater than those included in the study.

Additionally, enrollment in this study was hampered by other factors, such as the migrants’ difficulty in understanding the role of anthropologists in clinical intervention despite the explanation provided to them. Moreover, both the length of the questionnaire and the impact of the emotionally exhausting memories the interview generated probably limited enrollment.

Other critical factors included the fact that the simultaneous translation of the interview took time, and cultural mediators were sometimes excluded by choice of the individual migrant. This especially occurred when the cultural mediator and the interviewee spoke the same native language and/or came from the same country. We hypothesized that this depended on factors such as shame, fear of judgment, and lack of trust.

As shown in the tables, the participants did not answer all the interview items. This was either because the participants did not want to resume the interview after attending the center’s activities (e.g., medical check-ups, administration of topical therapy, interview with the police, the UNHCR, or the IOM, fingerprinting, meals, distribution of clothing and personal hygiene products) or because they could not, having been transferred to another facility in the national reception system. There was an awareness that the migrants who agreed to be interviewed may have done so because of the institutional role the researchers played, if only because the medical anthropologists wore the same uniforms as the doctors and nurses [40,42,43].

This means that the specificity of the research field not only hindered the complete collection of the data, but also influenced a higher recruitment. Indeed, the time and place of the anthropological study had to be coordinated with the participants’ other activities (as reported above). Once migrants were accepted into the center, their stay was regulated by timetables, scheduled medical appointments, police checks, and transfer to other facilities in the national reception system. In addition, when new irregular migrants landed on the island, all activities inside the center had to be suspended, with the exception of emergency medical care.

## 7. Conclusions

This study provides a description of the health needs of migrants that the healthcare and migrant reception systems should address. In particular, the collected information can be useful both in developing first aid interventions and in secondary healthcare support for migrants arriving by sea [37]. The ethnographic insight can both facilitate the identification of fragile health conditions and support clinicians in the diagnostic, therapeutic, and rehabilitation pathways.

This study’s results confirm that migration has a deleterious impact on health, especially in the event of cross-border displacements. In this regard, the qualitative data show that the participants were in good health when they left their country of origin and that their overland migration journey had a very negative impact on their health because of the violence and torture they underwent [4,12].

The opportunity to conduct medical anthropological research inside a first aid center permitted us to investigate migration at the end of the journey and to study the reception process on arrival.

## Figures and Tables

**Table 1:** Classification of referral violence and torture.

Group	Subgroup
Physical	Blunt trauma (punching, kicking, slapping, whipping, beating with wire rope, batons, throwing victim to the ground)
	Positional torture (suspension, stretching of limbs, prolonged restriction of movement, forced positioning)
	Burns from cigarettes, red-hot instruments, burning liquids, caustic substances
	Electric shocks
	Asphyxiation (by dry or water methods, drowning, suffocation, choking or use of chemicals)
	Crushing injuries (of fingers, use of heavy roller to crush thighs or back)
	Penetration wounds (knife, firearm, metal wires under fingernails)
	Exposure to chemical agents (salt, pepper, petrol, etc.) in wounds or body cavities
	Pharmacological torture (toxic doses of sedatives, neuroleptics, paralyzers, etc.).
	Crushing injuries or traumatic removal of limbs or fingers
	Surgical amputation of fingers or limbs, surgical removal of organs
Sexual	Sexual violence (blows to the genitals, harassment, use of instruments, rape)
Detention conditions	Detention conditions (in small or overcrowded cells, cell isolation, unhygienic conditions, lack of access to toilets, insufficient or infected food or water, exposure to extreme temperatures, denial of privacy rights and enforced nudity)
Sensory deprivation	Deprivation of normal sensory stimulation (sound, light, sense of time, isolation, manipulation of cell lighting, abuse in relation to physiological needs, restriction of sleep, food, water, access to health services and toilets, motor activities, medical care, social contact, isolation within the prison, loss of contact with the outside world)
Threats	Humiliation such as verbal abuse, performance of humiliating acts
	Threats of death, harm to family members, further torture, imprisonment, simulation of executions
	Threats of attacks by animals (cats, dogs, rats, scorpions)
Psychological	Psychological techniques to destroy the victim’s personality (forcing them to betray, confronting them with their powerlessness, exposing them to ambiguous situations or contradictory messages)
	Violation of taboos
	Behavioral coercion (engaging in practices contrary to the subject’s religion, coercion to harm others through torture or other abuse, coercion to destroy property, betray someone, and put their safety at risk)
	Forced to witness torture or atrocities inflicted on others

**Table 2:** Classification of the reported reasons for migration.

General	Detailed
Economic factors	Family conflicts (inheritance and land ownership)
	Poverty
	Unemployment
	Loss of family and social network support
	Absence of a welfare state
	Health problems
International protection	War
	Corruption of top politicians and security services
	Unfair judicial system
	Prisons
	Forms of violence/torture inflicted on the person
	Political persecution
	Lack of respect for human rights and/or freedom in general
	Other forms of persecution (specific)
	Lack of personal security

**Table 3:** Classification of the reported migration routes by the macro areas and countries crossed.

Macro Area	No.	%	Countries	No.
North Africa	22	22.2	Egypt, Libya	5
			Libya	12
			Tunisia, Libya	5
Central-North Africa	24	24.2	Niger, Libya	19
			Niger, Chad, Libya	1
			Sudan, Niger, Libya	4
East-North Africa	30	30.3	Egypt, Sudan, Libya	1
			Sudan, Libya	21
			Ethiopia, Sudan, Libya	8
West-Central-North Africa	5	5.1	Senegal, Mali, Burkina Faso, Niger, Libya	4
			Senegal, Mali, Niger, Libya	1
West-North Africa	12	12.1	Benin, Niger, Libya	1
			Burkina Faso, Niger, Libya	2
			Ghana, Togo, Burkina Faso, Niger, Libya	1
			Guinea, Mali, Burkina Faso, Niger, Libya	4
			Mali, Ivory Coast, Libya	1
			Mali, Niger, Libya	2
			Senegal, Algeria, Libya	1
Asia-Africa	6	6.1	Dubai, Jordan, Libya	1
			Jordan, Egypt, Libya	1
			Syria, Turkey, Libya	1
			Syria, Jordan, Sudan, Chad, Libya	1
			Sudan, Libya, Egypt, Israel, Rwanda, Uganda	1
			Malaysia, Uganda, Sudan, Chad, Libya	1
Total	99	100	Total	99

**Table 4:** Self-perceived health status by the stage of the migration route (107/112 respondents).

Self-Perceived Health Status	Country of Origin	During the Journey	At Lampedusa Center
	%	%	%
Very bad	6.5	66.7	11.2
Bad	29.9	23.1	35.5
Good	61.7	10.2	53.3
Very good	1.9	0.0	0.0
Total	100	100	100

**Table 5:** Reasons for seeking medical attention at the CPSA (71/112 respondents).

Reason	No.	%
Symptom from unspecified cause	15	21.1
Symptom from migratory journey	46	64.8
Symptom from CPSA	4	4.2
Medical need without symptoms	3	7.1
Symptom already in country of origin	9	12.7
Requested family visit	7	9.9
Symptom not reported	2	2.8
Total	71	100

**Table 6:** Type of violence and torture suffered (112/112 respondents).

Type	No.	%
Physical	57	50.9
Sexual	5	4.5
Detention conditions	37	33.0
Threats	29	25.9
Sensory deprivation	9	8.0
Psychological	54	48.2
Total	112	100

## Appendix A. Questionaries Interviste Etnografiche

**Data di compilazione:**

**Antropologa:**

**Mediatore transculturale:**

**Scheda Anagrafica**

Genere: M □ F □

Data di nascita (gg/mm/aaaa):

Scolarità: □ Fino a 5 anni di studio

□ Da 6 a 8 a nni di studio
□ Da 9 a 13 anni di studio
□ Da 14 a 19 anni di studio
□ Oltre 20 anni di studio

Data di arrivo sull’isola (gg/mm/aaaa):

Paese di origine:

Stato civile: Celibe □ Nubile □ Coniugato/a □ Separato/a □ Vedovo/a □

(1) Ragioni emigrazione e percorso migratorio
  1. Data di partenza dal proprio Paese di origine:
  2. Perché hai lasciato il tuo Paese di origine?
    □ Guerra
    □ Conflitti familiari
    □ Povertà
    □ Disoccupazione
    □ Perdita del sostegno della rete familiare e sociale
    □ Corruzione dei vertici politici e dei servizi di sicurezza
    □ Sistema giudiziario iniquo
    □ Assenza di un Welfare State (scuola, ass. medica, ass. sociale, servizi per l’impiego, sussidi per basso reddito, affido case popolari, sostegno alla nascita, ecc.)
    □ Carcerazioni
    □ Forme di violenza/tortura inferta alla persona
    □ Persecuzione politica
    □ Assenza del rispetto dei diritti umani e/o della libertà in generale
    □ Altre forme di persecuzione (specifica)
    □ Problemi di salute
    □ Insicurezza personale generalizzata
  3. Motivazione della partenza dal paese di origine rilevata dall’antropologa (si fa riferimento alle risposte della domanda n. 2):
  4. Nel tuo Paese ti sono state inferte forme di violenza? Sì □ No □
  5. Se sì, quali?
  6. Nel tuo Paese ti sono state inferte forme di tortura? Sì □ No □
  7. Se sì, quali?
  8. Nel tuo Paese, sei stato in carcere? Sì □ No □
  9. Se sì, per quanto tempo?
  10. Hai organizzato il viaggio da solo oppure altri lo hanno fatto per te? Da solo □ Altre persone □ Da solo e per mezzo di altre persone □
  11. Quanto è costato il viaggio?
  12. Quali Paesi hai attraversato?
  13. Dove hai dormito e mangiato nel corso del viaggio?
  14. Nel corso del viaggio hai subito violenza da parte di polizia/militari/trafficanti? Sì □ No □
  15. Se sì, ti hanno inferto forme di violenza? Sì □ No □
  16. Se sì, quali?
  17. Se sì, ti hanno inferto forme di tortura? Sì □ No □
  18. Se sì, quali?
  19. Nel corso del viaggio sei stato fermato da altri corpi di polizia/militari/trafficanti? Sì □ No □
  20. Se sì, ti hanno inferto forme di violenza? Sì □ No □
  21. Se sì, quali?
  22. Se sì, ti hanno inferto forme di tortura? Sì □ No □
  23. Se sì, quali?
  24. Sei stato trattenuto in carcere o in altra struttura detentiva? No□ Sì, in carcere □ Sì, in altra struttura □
  25. Se sì, dove e per quanto tempo?
  26. Durante la permanenza in carcere, o in altra struttura detentiva, ti hanno inferto forme di violenza? Sì □ No □
  27. Se sì, quali?
  28. Durante la permanenza in carcere, o in altra struttura detentiva, ti hanno inferto forme di tortura? Sì □ No □
  29. Se sì, quali?
  30. In quale porto di sei imbarcato e quando per raggiungere l’Isola di Lampedusa?
(2) Percezione del proprio stato di salute psico-fisica
  1. Prima di lasciare il tuo Paese di origine eri affetto da qualche malattia? Sì □ No □
  2. Se sì, quale
  3. Attualmente, com’è lo stato della tua salute? Pessimo □ Cattivo □ Buono □ Ottimo □
  4. Rispetto a quando sei partito dal tuo Paese il tuo stato attuale di salute è: Peggiore □ Uguale □ Migliore □
  5. Rispetto a quando ti sei imbarcato per Lampedusa il tuo stato di salute è: Peggiore □ Uguale □ Migliore □
  6. Che significa per te “essere in buona salute”?
  7. Durante la tua permanenza nel CSPA, dopo il controllo avvenuto al tuo arrivo, ti sei rivolto ancora ai medici? Sì □ No □
  8. Se sì, indica il motivo
(3) Progetto migratorio e aspettative
  1. Prima di partire dal tuo Paese, avevi un’idea rispetto a dove saresti emigrato? Sì □ No □
  2. Specifica la città/Paese/continente:
  3. Se hai risposto con una meta specifica, motiva:
  4. Hai membri della tua famiglia che sono rimasti nel tuo Paese di origine? Se sì, chi? □ Coniuge □ Figli □ Genitori/suoceri □ Nonni □ Fratelli/sorelle □ Cugini □ Zii
  5. Alcuni membri della tua famiglia sono qui con te? Se sì, chi? □ Coniuge □ Figli □ Genitori/suoceri □ Nonni □ Fratelli/sorelle □ Cugini □ Zii
  6. Hai membri della tua famiglia che vivono già in Europa? Se sì, chi e dove? □ Coniuge □ Figli □ Genitori/suoceri □ Nonni □ Fratelli/sorelle □ Cugini □ Zii
  7. Se hai familiari che già vivono in Europa, conti di fare affidamento a loro nel tuo progetto migratorio? Sì □ No □
  8. Che cosa ti aspetti da questo progetto migratorio?
  9. Quanto tempo pensi che durerà questo tuo progetto migratorio?
    □ Un anno
    □ Qualche anno
    □ Molti anni
    □ Per sempre
    □ Non so
  10. Torneresti indietro adesso? Sì □ No □ Non so □
  11. In futuro, pensi di tornare nel tuo Paese? Sì □ No □ Non so □
(4) Percezione del Sistema di Accoglienza italiano messo in atto nel CSPA sul progetto migratorio
  1. Cosa immaginavi di trovare al tuo arrivo in Italia?
  2. C’è stata qualcosa che ti ha colpito particolarmente, sia in senso positivo che negativo, quanto sei stato soccorso in mare, quando sei arrivato al porto, quando sei entrato nel CSPA? Sì □ No □ Non so □
  3. Se sì, specificare:
  4. Pensi che queste cose che ti hanno colpito potranno influenzare la realizzazione del tuo progetto migratorio? Sì □ No □ Non so □
  5. Pensi che in Italia, oppure dove hai pensato di migrare, potrai realizzare il tuo progetto migratorio? Sì □ No □ Non so □
  6. Che significato ha il numero che ti hanno dato all’arrivo al centro, secondo te?
  7. Ti è mai capitato di perderlo o di scambiarlo con quello di un altro? Sì □ No □
  8. Se si, motivalo:
